# An Evaluation of Longitudinal Thyroid Hormone Levels Over the Years Before and After Patients Started on Levothyroxine

**DOI:** 10.1002/edm2.70214

**Published:** 2026-04-07

**Authors:** Michael Stedman, Peter Taylor, Ian Halsall, David Halsall, Buchi Okosieme, Suhani Bahl, Sangeeth Veluchamy, Lakdasa Premawardhana, Anthony A. Fryer, Colin Dayan, Adrian Heald

**Affiliations:** ^1^ Res Consortium Andover UK; ^2^ Cardiff University School of Medicine Cardiff UK; ^3^ Addenbrooke's Hospital, Cambridge UK; ^4^ University College, London UK; ^5^ Keele University Keele UK; ^6^ University of Manchester Manchester UK; ^7^ Salford Royal Hospital Salford UK

**Keywords:** cohort, hypothyroidism, levothyroxine, freeT4, TSH, retrospective, treatment

## Abstract

**Introduction:**

This retrospective study aimed to investigate how free thyroxine (FT4) and thyroid‐stimulating hormone (TSH) levels change in the years pre‐ and post‐initiation of levothyroxine (LT4). A better understanding of the relation between thyroid hormone replacement therapy and actual levels of TSH/FT4 in patients could allow more tailored and effective therapy for people with hypothyroidism.

**Methods:**

Using de‐identified citywide population health records, we were able to analyse TSH and FT4 results during the period 2012–2023 (12 years) for patients whose LT4 was started during 2015–2019 (5 years) and who also had a diagnosis recorded of primary hypothyroidism.

**Results:**

Before LT4 initiation, there was a slight rise in median TSH results over the previous years with a sharper increase in the 12 months prior to medication starting. Once on medication, TSH fell to levels significantly below the pre‐medication levels. Between −6 and −2 years before initiation of LT4, median TSH increased from 4.0 to 4.9 mu/L and then in the last year before initiation to 6.4 mu/L. Once on medication the TSH fell to 3.8 mU/L then to 2.7 mU/L over subsequent years, which was significantly below the TSH level before LT4 prescribing was initiated. Median FT4 was low and falling slowly prior to medication starting. Once medication started, FT4 was higher year on year, in relation to increasing doses of LT4 with TSH correspondingly lower. Average daily LT4 dose continued to increase over the years following initiation, with a corresponding rise in FT4. The median LT4 dose for the treated group rose steadily from 49mcg to 69mcg daily.

**Conclusion:**

This population‐based retrospective study reflects varying responses in different patients, but with overall further evidence for increasing difference over time in FT4 and TSH in treated hypothyroid individuals versus euthyroid individuals. This has implications for how LT4 dose titration is conducted in treated hypothyroidism.

## Graphical abstract

Free thyroxine (FT4) median (interquartile range) and percentage FT4 within reference range for patients pre‐ and post‐levothyroxine (LT4) treatment versus untreated individuals.

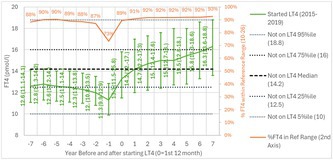

## 1 Introduction

There continues to be much discussion around optimization of thyroid hormone status in people treated for hypothyroidism [1]. The ideal therapeutic goal in hypothyroidism would be restoration of clinical and biochemical euthyroidism with thyroid hormone replacement although there is accumulating evidence of a very different thyroid hormone profile in treated hypothyroid individuals versus euthyroid individuals [2]. Free triiodothyronine (FT3) is sometimes included, supported by guidance [3], particularly in people taking combination levothyroxine (LT4) and liothyronine (LT3) therapy [4].

The signs and symptoms that suggest thyroid dysfunction are non‐specific and non‐diagnostic, especially early in presentation. Diagnosis is therefore based on blood levels of thyroid‐stimulating hormone (TSH) and free thyroxine (FT4). Symptom relief and normalised TSH levels are achieved with LT4 replacement therapy, started at 1.5–1.8 mcg per kg per day (recommended) [5].

A sensitive negative feedback loop regulates the thyroid hormone production. The hypothalamus produces thyrotropin‐releasing hormone (TRH) that controls anterior pituitary gland secretion of TSH, regulating the secretion of the thyroid hormones FT3 and FT4 by the thyroid gland [6, 7]. FT4 and FT3 affect the metabolism and function of many cells and organs. Thyroid hormones also regulate thyroid metabolism by providing negative feedback to the hypothalamus and pituitary gland. The hypothalamus adjusts the release of TRH based on circulating levels of thyroid hormone.

The pathophysiology of autoimmune hypothyroidism is generally characterised by the presence of thyroid peroxidase and thyroglobulin antibodies, with an associated infiltration of lymphocytes in the thyroid. Due to the progressive destruction of cells, subclinical or overt hypothyroidism can ensue. The pathophysiological process involves the combination of genetic, environmental and epigenetic factors [8].

In recent study [9], growth mixed model (GMM) analysis was used to model TSH trajectory during LT4 treatment. In the Brazilian study population, the investigators identified four distinct TSH trajectory classes. The classes were identified by the path of the TSH trajectory relative to the normal reference range. The baseline LT4 dose emerged as a key difference between the classes. In a separate analysis, Devdhar et al. in a retrospective notes‐based study found that LT4 replacement doses were affected by sex and weight, but not age [10]. Other studies have also examined the equilibria between free thyroid hormones and TSH and the way that the equilibrium is modulated by various influences including age, body mass index and treatment [11, 12].

Resolution of symptoms can be documented even with treatment of mild hypothyroidism [13], but few studies document longitudinal monitoring of both symptoms and biochemical parameters within individual treated patients [14]. A better understanding of the relation between thyroid hormone replacement therapy and circulating levels of TSH and FT4 in patients could allow more tailored and effective therapy for people with hypothyroidism as recommended in American Thyroid Association guidance [14].

Long‐term population studies have demonstrated that the distribution of TSH shifts with age and differs between individuals with and without thyroid disease. Follow‐up of the Wickham cohort [15], data from the Norwegian HUNT study [16] and the Busselton Health Study [17] all show a progressive rightward drift of TSH with ageing in otherwise healthy populations. These observations highlight that reference‐range‐based interpretation does not fully capture physiological set‐point variation over time and provide important context when interpreting TSH behaviour in treated hypothyroidism over many years.

Additionally, multiple studies [18, 19] suggest that between 20% and 40% of patients taking LT4 have TSH levels outside of the normal range, indicating potential under‐ or over‐replacement of thyroid hormones. We and others have reported that the distribution of FT4 versus TSH was markedly different in people with treated hypothyroidism versus people with euthyroidism at the time of their thyroid function tests (TFTs) being checked [2, 18, 19, 20].

In this study we investigate how TFTs—FT4 and TSH change in the years pre‐ and post‐initiation of LT4 replacement over a period of up to 8 years post‐initiation of LT4. We mapped within‐person trajectories before/after LT4 start. We quantified long‐term dose drift and FT4/TSH shifts over time.

## 2 Methods

Using citywide population health records, we were able to analyse TSH/FT4 results during the period 2012–2023 (total 12 years) for de‐identified patients diagnosed with primary hypothyroidism. All patients included had a primary thyroid diagnosis of autoimmune hypothyroidism.

For the purpose of this analysis the focus was on patients whose medication started within the period 2015–2019 (5 years) and so their TFT results pre‐ and post‐LT4 initiation could be investigated. The study was also reviewed and approved by the Greater Manchester Care Record (GMCR) Expert Research Group (ERG) reference number R 2023 065 [21]. The data used in the analyses presented was obtained with the permission of the GMCR Board ERG (reference number R 2023 065) and was fully deidentified prior to being made available to the investigators.

Data were retrieved from a pooled general practitioner research database covering the whole conurbation. All patients on LT4 with a coded diagnosis of autoimmune hypothyroidism (ICD‐10 or SNOMED codes) [22, 23] were included. The diagnoses of central hypothyroidism, post‐thyroid cancer suppression, postpartum thyroiditis, lithium/amiodarone exposure, pregnancy and transitory thyroiditis were excluded.

FT4 and TSH assays were performed using the Siemens Atellica IM immunoassay (Munich, Germany) platform across the laboratories contributing to the GMCR. All laboratories participate in regular UK NEQAS external quality assurance programmes, minimising inter‐laboratory and temporal assay drift. A conservative reference range for FT4 (10–26 pmol/L) was adopted to encompass the ranges used across all participating laboratories during the study period.

As FT4 and TSH are physiologically linked through hypothalamic–pituitary feedback, composite indices (FT4/TSH and FT4 × TSH) have previously been used to explore altered homeostatic equilibria in LT4‐treated patients compared with untreated individuals [11, 12]. In this study, these indices were examined descriptively as exploratory physiological markers rather than as validated clinical indices.

We compared results with patients for whom:

the primary diagnosis was hypothyroidism,

they had more than 5 years of LT4 prescribing records and

TFT were recorded over more than 5 years, of which at least 2 years were prior to medication starting.

Results were compared with the results from patients who had no record of LT4 prescribing at any point and no diagnosis of thyroid disease.

Prescribing data was aggregated to provide total mcg of LT4 prescribed in each year for each patient. Year of birth data was applied to calculate the patient's age at the start of LT4 medication and the patient's age at the time of TFT testing.

Reference ranges relevant to diagnosis were TSH minimum 0.5mu/L, 5.0 mU/L and FT4 10.0–25.0 pmol/L. Due to the skewed distribution of TSH, especially in the treated patients, the median value and interquartile range (IQR) across the population in each year was used for examining how these indicators developed over time.

Further examination over the period before and after the start of medication included change in FT4/TSH ratio and product FT4xTSH and the median of the patient's annual average daily dose in each of the years after the start of medication.

Thyroid hormone profile in sub‐populations was also examined by splitting out

- patient's average daily dose over their whole prescribing period into those on low (0–39mcg/day), moderate (40–79mcg/day), high (80–120mcg/day), and higher (120+ mcg/day) daily doses,
- age at start of medication (< 60 years and 60+ years) and
- sex (male and female).

### 2.1 Statistics

This analysis was conducted by aggregating and filtering the large annual csv files using Delimit software (Microsoft Windows software tool) and then analysing the consolidated 2010–2023 data using Excel Power Pivot. This was an observational analysis, so statistical analysis was kept to a minimum and no correlations were calculated. Medians were used as the distribution of TSH was not normal and IQRs have been added. As the cohort varies in each year in relation to the precise numbers of patients, techniques used such as Cox regression were not carried out.

## 3 Results

The GMCR provided 12.8 million TFT results for 1.13 million people from 2005 to 2024. Patient diagnosis and medication prescription data were also used (Table 1) to assemble the 146,686 TFT results for 6224 patients whose primary diagnosis was autoimmune hypothyroidism, who started LT4.

**TABLE 1:** The number of patients tested and the number of TFTs carried out with median age (IQR) at the time of the test and median (IQR) TSH mu/L and FT4 pmol/L values recorded.

		Number of patients	Total TFTss	Median age (IQR)	% Females	Median TSH/%iRR (IQR)	Median FT4/%iRR (IQR)
Total tested		1,137,092	12,802,025	55 (41–67)	65.7%	1.76/84% (1.1–2.8)	14.4/92% (12.5–16.6)
Overall 2012–2023		1,088,209	9,284,536	56 (41–69)	64.6%	1.75/85% (1.1–2.8)	14.3/94% (12.5–16.3)
Of which	No diagnosis/No LT4	943,201	6,559,531	54 (39–68)	58.1%	1.7/95% (1.2–2.4)	14.2/96% (12.5–16.)
	Hypothyroidism diagnosis + LT4	89,859	1,859,892	60 (48–72)	81.2%	2.4/61% (0.9–4.6)	15.1/89% (12.4–18.3)
	Thyroid disorder diagnosis	38,453	649,536	57 (44–72)	81.4%	1.5/55% (0.4–3.5)	15.8/85% (12.8–19.4)
Of those diagnosed with hypothyroidism and treated with LT4							
Started LT4 in 2015_19		18,245	351,036	53 (40–66)	77.8%	3.5/58% (1.8–6.0)	13.7/86% (11.3–16.7)
Of whom had 5+ years of medication		13,078	269,349	53 (40–66)	79.0%	3.4/58% (1.6–5.9)	13.8/86% (11.4–16.9)
Of whom had tests both in > 5 years in total and in > 2 years before medication		6224	146,686	55 (43–67)	78.0%	3.7/60% (2.1–5.8)	13.2/87% (11.2–16.0)

This analysis shows for the key groups:

- 943,201 population who had no diagnosis or LT4 medication had 6,559,531 TFTs within the 12 years; they were 58% female with median age of 54 years at time of testing and results were median TSH 1.7 mU/L (IQR 1.2–2.4) with 95% of results within reference range while median was FT4 14.2 pmol/L (IQR 12.5–16.0) with 96% of results within reference range.
- 89,859 had a diagnosis of hypothyroidism and were being treated with LT4. They had 1,859,892 TFTs with a median age of 60 and they were 81% female with a median age of 60. Median TSH was 2.4 mU/L with 61% within reference range and median FT4 was 15.1 pmol/L with 89% within reference range.
- 18,245 were started on LT4 during the period 2015–2019; they had a total of 351,036 TFTs, with median age 53% and 78% were female. The median TSH was 3.5 mU/L with 58% of results within reference range and median FT4 at 13.7 pmol/L with 86% within reference range. Compared with those already on LT4 medication, new patients to LT4 had a higher % males and were of younger age.
- The 6224 patients who had sufficient time on medication LT4 and TFT results to be included into this longitudinal analysis had 146,686 TFT results; median age of 55, with 78% female, with TSH 3.7 mu/L and 60% in reference range, and FT4 13.2 pmol/L and 87% within reference range.

**FIGURE 1:**
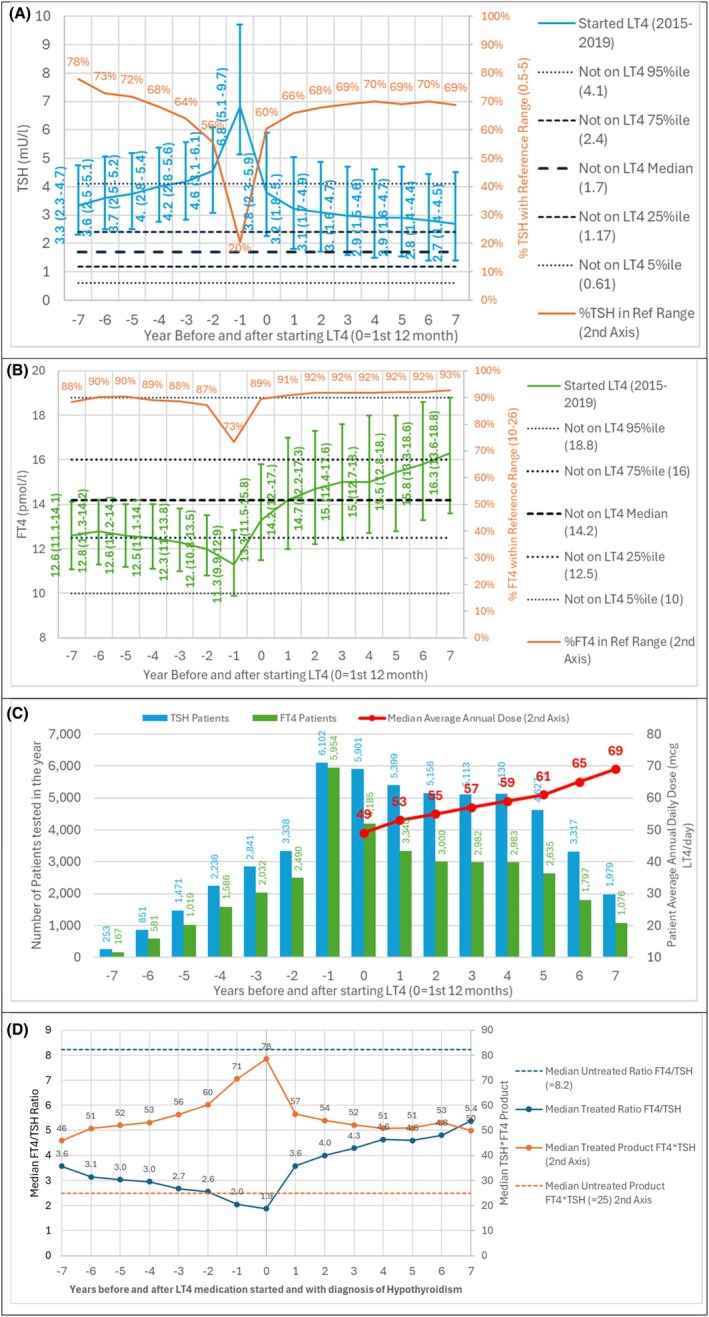
TFT result development in the years before and after starting LT4 compared with the untreated population median 5%, 25%, 75% and 95% values (A) TSH median (IQR) and % within reference range (0.5–5.0 mU/L), (B) FT4 median (IQR) and % within reference range 10–25 pmol/L. (C) Number of patients tested and median annual LT4 dose mcg/patient/day and (D) ratio and product of FT4 and TSH.

Figure 1 shows development of TFT results before and after starting LT4. Figure 1A shows the median TSH increasing in the 6 years prior to LT4 initiation from 3.6 mU/L to 4.6 mU/L, with a sharp increase to 6.8 mU/L in the year immediate prior to medication starting. Once on medication the TSH fell to 3.8 mU/L then to 2.7 mU/L over subsequent years, which was significantly below the TSH level before LT4 prescribing was initiated. The % within reference range fell from 78% to 56% over the 6 years, with a sharp fall to 20% in the year before medication. The % rose once medication had started; however, it only reached 70% within reference range even after 6 years—this is well below the 95% achieved by untreated patients.

Figure 1B shows the median FT4 falling steadily in the 6 years prior to LT4 initiation from 12.8 pmol/lL to 12.0 pmol/L, with a slightly larger fall to 11.3 pmol/L in the year prior. Once medication started, the median FT4 increased year on year, reaching 16.3 pmol/L after 7 years. The % within FT4 reference range (10–25 pmol/L) fell slightly from 90% to 87%, falling to 73% in the year before medication; it then increased to 89% immediately after medication started, reaching 93% after 7 years—this compares with 96% in the non‐medicated population.

Figure 1C shows the numbers of patients within the selected group being tested for TSH and FT4 in each year rising to 6102 in year before medication, 98% of those selected. The median average annual daily dose being prescribed across the group also showed a steady rise from 49mcg/day in the first year to 69mcg/day, which could be the source of the increase in median FT4 over the same period.

Figure 1D shows using individual TFT results; the ratio FT4/TSH was below and the product T4/TSH was above individuals ‘categorised’ as euthyroid—suggesting that the balance between FT4 and TSH as reflective of the regulation of TSH at a hypothalamic/pituitary level was different in treated versus untreated individuals.

**FIGURE 2:**
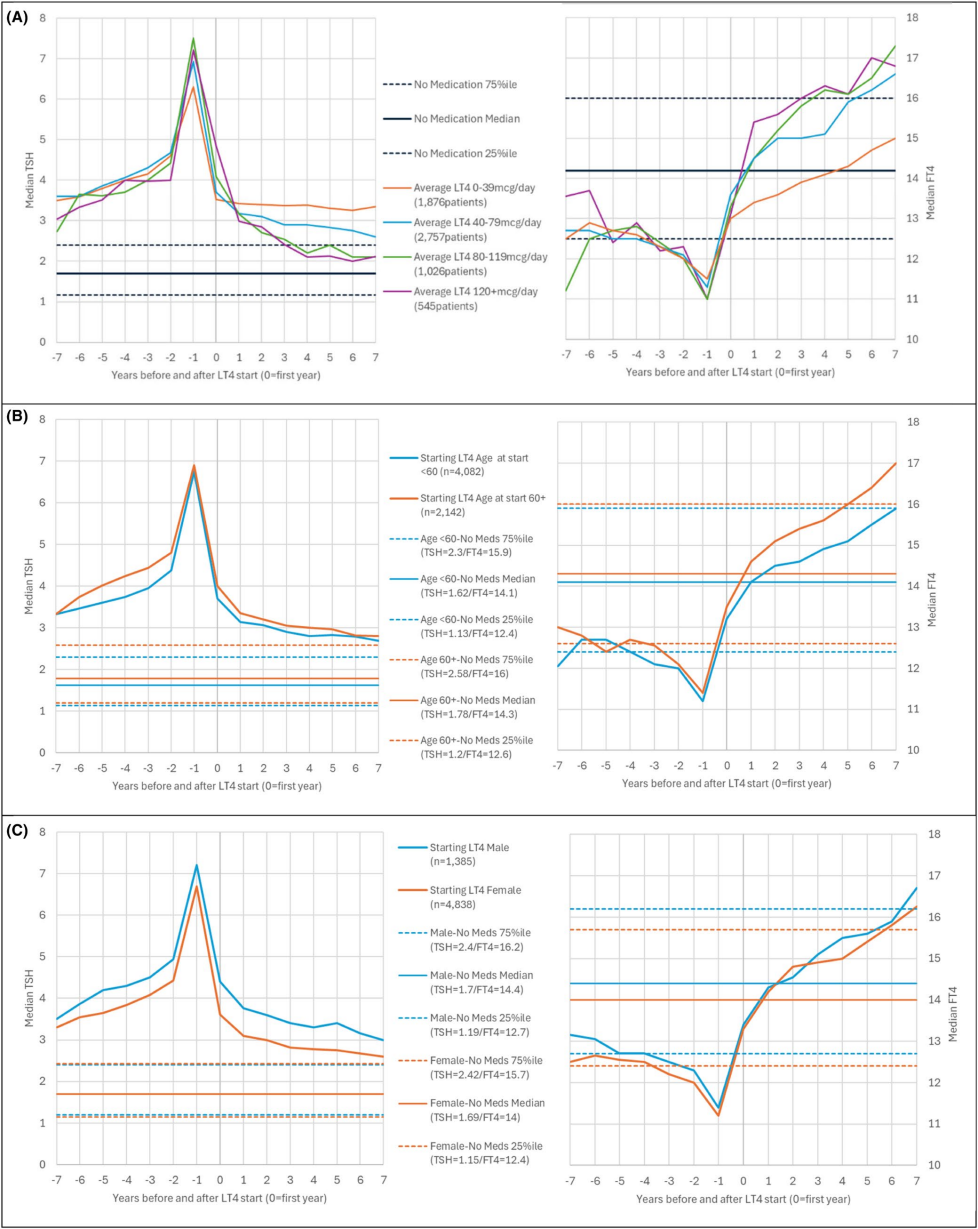
Median FT4 (on the right hand side) and TSH (on the left hand side) for the years before and after starting treatment with LT4, compared with the median and IQR for the equivalent untreated population split by (A) average daily dose < 40mcg/40–79mcg/80+ mcg (B) age at start of LT4 < 60 and 60+ years and (C) sex (male vs. female).

Figure 2 reflects the trend in TSH and FT4 when the treated cohort is split by average daily dose, age and sex. Figure 2A shows little difference in FT4 and TSH levels by dose before medication started. However, once on medication, the lower dose patients show higher TSH and lower FT4 results, with those patients on 40+ mcg/day median increasing to above the euthyroid 75 percentile for FT4. Figure 2B shows that median FT4 in the older age 60+ group also increased to above the 75 percentileile for the untreated older patients. Figure 2C shows little sex difference. However, this indicates that males chosen for LT4 treatment might have had higher TSH than the females before and after treatment.

## 4 Discussion

This retrospective data‐based study reflects varying responses in different individuals. In the context of the decrease in median TSH over time, measured FT4 continued to increase significantly in the years after the medication started, suggesting that dose for a different balance between FT4 and TSH in treated hypothyroid versus untreated individuals. These findings relate to the population of Greater Manchester, UK as treated in primary care in relation to FT4 and TSH laboratory results and LT4 prescriptions [20]. Median age of the untreated individuals and those on LT4 selected for analysis was similar with a higher proportion of women (78% vs. 58%) in the treated versus untreated group.

It is also the case that TFT testing is not always carried out according to guidance in relation to test intervals with marked inter‐general practice variability [24]. Furthermore, some individuals with a normal TSH were started on LT4.

As shown in Figure 1C, the ratio FT4/TSH was below and the product T4/TSH was above individuals ‘categorised’ euthyroid—suggesting a situation in which the balance between FT4 and TSH as reflective of the regulation of TSH at a hypothalamic/pituitary level was different in treated versus untreated individuals. This has been described previously by Hoermann et al. in 2013 and 2014 [11, 12]. The authors of the 2014 paper [12] reported in a prospective observational study that in LT4‐treated patients, the equilibria involving FT3, FT4 and TSH differed markedly from those in untreated individuals. We previously described this in a cross‐sectional analysis published recently, where only at a FT4 level of 20 pmol/L was there confluence between FT4/TSH relation between treated and untreated individuals [20].

In relation to the dose of LT4 post‐initiation of LT4, the median dose was quite low in relation to the recommended dose of 1.6 mcg/kg [25]. Analysis of the actual doses prescribed suggests that under 49% of people were being prescribed more than 50 mcg of LT4 in the first year after diagnosis, with this increasing to 63% by the fifth year after diagnosis.

As might be expected, FT4 levels were higher and TSH lower as the dose range of LT4 rose (Figure 2A). Importantly, the FT4 levels were well above even the 75th centile for the untreated population.

The higher levels of FT4 and of TSH in older versus younger patients were seen in our earlier smaller study [2]. It is well known that TSH levels rise with age in older individuals with an increasing trend with age. A ‘natural’ trend for TSH to increase with advancing years has been documented in the older population, even in subjects with no documented thyroid disease [26]. We report here similar findings in older people with treated hypothyroidism of 60+ years versus younger individuals. A key question is to what degree clinicians should aim for a reference range TSH in older people, particularly those over the age of 80 years who are taking LT4, given the potential cardiac adverse effects of overtreatment hypothyroidism in this group [27].

The consistently higher TSH in women versus men at all ages in treated individuals is intriguing. It certainly seems to be the case that thyroid hormone physiology is rebalanced during LT4 monotherapy, compared with the physiological state. The newly formed equilibria seem to maintain a centrally overcompensated and peripherally undercompensated state, with TSH levels partly suppressed [9].

In individuals with persistently elevated TSH despite an apparently adequate replacement dose of LT4, poor compliance, malabsorption and the presence of drug interactions should be checked. Over‐replacement is common and is associated with increased risk of atrial fibrillation and osteoporosis: hence should be avoided [28].

Hypothyroidism affects nearly 2% of the population of the United Kingdom [29, 30], but as many as 10% of treated individuals are dissatisfied with LT4 monotherapy because of persistent symptoms and a poor quality of life (QOL) [31, 32]. For some people, response to levels of FT4 coming from prescribed LT4 may not be the same as for endogenous LT4 [33, 34], hence progressive LT4 dose escalation by prescribers for some and the addition of LT3 for others [35].

Interpretation of TFTs in LT4 treated individuals should consider not only under‐replacement but also the potential for over‐replacement in relation to laboratory reference ranges.

Notable following LT4 initiation, median FT4 values in our cohort progressively rose above the untreated population median and approached the upper centiles of the reference distribution over time. This suggests that, despite TSH normalisation, circulating FT4 levels may enter a range that would be considered relatively high in untreated individuals. We suggest that reference ranges derived from untreated populations may not fully reflect the physiological state of patients receiving LT4. Furthermore, there may be small but clinically significant differences between assay reference ranges [36]. Progressive dose escalation may result in FT4 levels that sit towards the upper end of, or above, the typical physiological distribution. This is particularly relevant in older individuals where overtreatment carries recognised risks [26, 27].

We speculate that for some people peripheral and central tissue response to levels of FT4 coming from prescribed LT4 are not the same as for endogenous LT4, hence progressive dose escalation over time for some.

From a practical perspective, these findings suggest that clinicians should be cautious about progressive LT4 dose escalation in response to modest TSH elevations over time, particularly in older individuals. Our data show that FT4 levels may rise progressively following LT4 initiation despite TSH remaining within the reference range, potentially leading to relative over‐replacement when judged against physiological distributions seen in untreated populations. In older patients, where the risks of overtreatment are well recognised, this may warrant a more conservative approach to dose adjustment and closer biochemical follow‐up rather than reliance on TSH alone. These observations support careful interpretation of TFTs during long‐term LT4 therapy and suggest that stable, mildly elevated TSH values in older individuals may not always necessitate dose escalation.

It may be that there is a testable biochemical explanation that low‐dose LT4 administration reduces TSH short term, thereby further shrinking functional gland sizes and, as a consequence, leads to a rise in TSH long term to rebalance the system.

Regarding limitations, we accept that the data was taken from one conurbation only and was retrospective in nature. The evidence class is low and should not be overstated. We accept that the individuals were not all the same in each year examined—however most were followed through year on year. Regarding FT3, FT3 levels are not checked as part of routine care for hypothyroidism in the United Kingdom. We only have FT3 data on a small proportion of patients who are likely not to be representative of the larger group. Therefore, FT3 was not included in the analysis. Also, we did not have access to non‐coded data including symptomatic responses to treatment. This much be balanced against the fact that we accessed deidentified data from all general practices covering a city population of 2.85 million people. We accept that the composite indices FT4/TSH and FT4 × TSH presented here are exploratory physiological markers and are not established or validated clinical tools.

We were able to download all TFTs before and after the initiation of LT4 for a period of 5 years to include several thousand people. We also accept that there will be batch‐to‐batch variation in the assays for FT4 which may affect whether tests fall within or just outside any laboratory reference range. Nevertheless, by taking a conservative reference range for FT4 which encompasses all laboratory platforms in Greater Manchester, we have endeavoured as far as possible to address this matter. It is also the case that some people may be started on LT4 when more time might have been given to monitor thyroid hormone status before moving to LT4 replacement [37, 38].

This retrospective data‐based study reflects varying responses in different individuals. In the context of the decrease in median TSH over time, measured FT4 continued to increase significantly in the years after the medication started, suggesting that dose for a different balance between FT4 and TSH in treated hypothyroid versus untreated individuals. These findings relate to the population of Greater Manchester as treated in primary care in relation to FT4 and TSH laboratory results and LT4 prescriptions.

Three previous studies, the Wickham follow‐up cohort [15], the Norwegian HUNT study [16] and the Busselton Health Study [17] described the progressive rightward drift of TSH with ageing in otherwise healthy populations. This contextualises our findings, particularly in relation to the higher TSH values observed in older individuals both before and after LT4 initiation.

This population‐based study reflects varying responses in different patients, but with overall further evidence for the increasing difference over time in TFT profile in treated hypothyroid individuals versus euthyroid individuals, with trajectory tracing the years both before and after LT4 initiation. We accept that the data that we evaluated was taken from a single geographical region. However, we had access to all TFTs checked and the region is typical of the United Kingdom as a whole in terms of the demographic mix.

Both LT4 dose and measured FT4 continue to increase significantly in the subsequent years after the medication started, suggesting that the physiology underlying dose adjustments of LT4 is complex. There may be a testable biochemical explanation that low‐dose LT4 administration reduces TSH short term, thereby further shrinking functional gland sizes and, as a consequence, leading to a rise in TSH long term to rebalance the system.

In conclusion, this retrospective observational longitudinal study including a large sample from a UK city register shows on average progressive LT4 dose escalation over a 7‐year period. Together with accumulating evidence in the literature, despite some acknowledged limitations, this study supports the notion that central and peripheral responses to levels of FT4 are not the same in LT4‐treated patients and euthyroid subjects. This observation may have treatment implications that require further and more detailed study including correlation to patient symptoms.

One thing that seems clear is that the reality is different from guideline assumptions. A potential recommendation from this paper is that there is careful consideration of LT4 dose adjustment in people with hypothyroidism, as once the dose of LT4 is increased it is difficult to reduce it again. Furthermore, any adjustments in management need to take account of the age of the individual; the finding of varying FT4/TSH ratio over time underlies the principle that both FT4 and TSH should ideally be tested routinely in people with treated hypothyroidism [39].

## Author Contributions

**Anthony A. Fryer:** conceptualization, validation, writing – review and editing, project administration, supervision. **Buchi Okosieme:** writing – original draft, writing – review and editing, validation, supervision. **Colin Dayan:** conceptualization, investigation, writing – review and editing, visualization, validation, methodology, project administration, supervision. **Adrian Heald:** conceptualization, investigation, writing – original draft, methodology, validation, writing – review and editing, visualization, project administration, supervision, resources, data curation.

## Funding

The authors have nothing to report.

## Ethics Statement

The study was reviewed by the Greater Manchester Care Record (GMCR) Expert Research Group (ERG) reference number R 2023 065 who gave a favourable opinion. De‐identified data was used, as per the Health Research Authority's Governance arrangements and in accordance with the Declaration of Helsinki. Human Ethics and Consent to Participate declarations were not applicable.

## Conflicts of Interest

The authors declare no conflicts of interest.

## Data Availability

The data that support the findings of this study are available on request from the corresponding author. The data are not publicly available due to privacy or ethical restrictions.

## References

[1] B. Biondi and D. S. Cooper , “Thyroid Hormone Therapy for Hypothyroidism,” Endocrine 66, no. 1 (2019): 18–26. 31372822 10.1007/s12020-019-02023-7

[2] A. H. Heald , L. D. Premawardhana , P. N. Taylor , et al., “How Does Thyroid Hormone Profile Differ on and Off Replacement Treatment?,” Clinical Endocrinology 102, no. 4 (2025): 490–495. 39702980 10.1111/cen.15185 PMC11874186

[3] National Guideline Centre (UK) , Thyroid Function Tests: Thyroid Disease: Assessment and Management: Evidence Review C (National Institute for Health and Care Excellence (NICE), 2019). 35129920

[4] P. Saravanan , H. Siddique , D. J. Simmons , R. Greenwood , and C. M. Dayan , “Twenty‐Four Hour Hormone Profiles of TSH, Free T3 and Free T4 in Hypothyroid Patients on Combined T3/T4 Therapy,” Experimental and Clinical Endocrinology & Diabetes 115, no. 4 (2007): 261–267. 17479444 10.1055/s-2007-973071

[5] S. A. Wilson , L. A. Stem , and R. D. Bruehlman , “Hypothyroidism: Diagnosis and Treatment,” American Family Physician 103, no. 10 (2021): 605–613. 33983002

[6] R. M. Pluta , A. E. Burke , and R. M. Glass , “JAMA Patient Page. Subclinical Hypothyroidism,” Journal of the American Medical Association 304, no. 12 (2010): 1402. 20858886 10.1001/jama.304.12.1402

[7] J. W. Dietrich , G. Landgrafe , and E. H. Fotiadou , “TSH and Thyrotropic Agonists: Key Actors in Thyroid Homeostasis,” Journal of Thyroid Research 2012 (2012): 351864. 23365787 10.1155/2012/351864 PMC3544290

[8] E. Tywanek , A. Michalak , J. Świrska , and A. Zwolak , “Autoimmunity, New Potential Biomarkers and the Thyroid Gland‐The Perspective of Hashimoto's Thyroiditis and Its Treatment,” International Journal of Molecular Sciences 25, no. 9 (2024): 4703. 38731922 10.3390/ijms25094703 PMC11083198

[9] M. D. Ettleson , G. C. E. Penna , W. Wan , I. M. Benseñor , N. Laiteerapong , and A. C. Bianco , “TSH Trajectories During Levothyroxine Treatment in the Brazilian Longitudinal Study of Adult Health (ELSA‐Brasil) Cohort,” Journal of Clinical Endocrinology and Metabolism 109, no. 12 (2024): 3065–3075. 38780968 10.1210/clinem/dgae294 PMC11570358

[10] M. Devdhar , R. Drooger , M. Pehlivanova , G. Singh , and J. Jonklaas , “Levothyroxine Replacement Doses Are Affected by Gender and Weight, but Not Age,” Thyroid 21, no. 8 (2011): 821–827. 21751885 10.1089/thy.2011.0029 PMC3148125

[11] R. Hoermann , J. E. Midgley , R. Larisch , and J. W. Dietrich , “Is Pituitary TSH an Adequate Measure of Thyroid Hormone‐Controlled Homoeostasis During Thyroxine Treatment?,” European Journal of Endocrinology 168, no. 2 (2013): 271–280. 23184912 10.1530/EJE-12-0819

[12] R. Hoermann , J. E. Midgley , A. Giacobino , et al., “Homeostatic Equilibria Between Free Thyroid Hormones and Pituitary Thyrotropin Are Modulated by Various Influences Including Age, Body Mass Index and Treatment,” Clinical Endocrinology 81, no. 6 (2014): 907–915. 24953754 10.1111/cen.12527

[13] F. Monzani , N. Caraccio , P. Del Guerra , A. Casolaro , and E. Ferrannini , “Neuromuscular Symptoms and Dys‐ Function in Subclinical Hypothyroid Patients: Beneficial Effect of LT4 Replacement Therapy,” Clinical Endocrinology 51 (1999): 237–242. 10468996 10.1046/j.1365-2265.1999.00790.x

[14] J. Jonklaas , A. C. Bianco , A. J. Bauer , et al., “American Thyroid Association Task Force on Thyroid Hormone Replacement. Guidelines for the Treatment of Hypothyroidism: Prepared by the American Thyroid Association Task Force on Thyroid Hormone Replacement,” Thyroid 24, no. 12 (2014): 1670–1751. 25266247 10.1089/thy.2014.0028 PMC4267409

[15] M. P. Vanderpump , W. M. Tunbridge , J. M. French , et al., “The Incidence of Thyroid Disorders in the Community: A Twenty‐Year Follow‐Up of the Whickham Survey,” Clinical Endocrinology 43, no. 1 (1995): 55–68. 7641412 10.1111/j.1365-2265.1995.tb01894.x

[16] B. O. Asvold , T. Bjøro , C. Platou , and L. J. Vatten , “Thyroid Function and the Risk of Coronary Heart Disease: 12‐Year Follow‐Up of the HUNT Study in Norway,” Clinical Endocrinology 77, no. 6 (2012): 911–917. 22724581 10.1111/j.1365-2265.2012.04477.x

[17] A. P. Bremner , P. Feddema , P. J. Leedman , et al., “Age‐Related Changes in Thyroid Function: A Longitudinal Study of a Community‐Based Cohort,” Journal of Clinical Endocrinology and Metabolism 97, no. 5 (2012): 1554–1562. 22344200 10.1210/jc.2011-3020

[18] A. C. Bianco , Y. Bao , O. Antunez Flores , et al., “Levothyroxine Treatment Adequacy and Formulation Changes in Patients With Hypothyroidism: A Retrospective Study of Real‐World Data From the United States,” Thyroid 33, no. 8 (2023): 940–949. 37335236 10.1089/thy.2022.0382

[19] A. Efthymiadis , M. Henry , D. Spinos , et al., “Adequacy of Thyroid Hormone Replacement for People With Hypothyroidism in Real‐World Settings: A Systematic Review and Meta‐Analysis of Observational Studies,” Clinical Endocrinology 100, no. 5 (2024): 488–501. 38037493 10.1111/cen.14998

[20] A. H. Heald , L. D. Premawardhana , P. N. Taylor , et al., “Evaluating the Link Between Thyroid Function Test Results and Levothyroxine Dose in the Management of Hypothyroidism: Can we Improve Dosing Regimes?,” Clinical Endocrinology 104 (2025): 372–385, 10.1111/cen.70074. 41429661 PMC12954151

[21] https://healthinnovationmanchester.com/thegmcarerecord/the‐gm‐care‐record‐for‐secondary‐uses‐research. Accessed 21 June 2025.

[22] https://icd.who.int/browse10/2019/en. Accessed 21 June 2025.

[23] https://digital.nhs.uk/services/terminology‐and‐classifications/snomed‐ct. Accessed 21 June 2025.

[24] J. J. Scargill , M. Livingston , D. Holland , C. J. Duff , A. A. Fryer , and A. H. Heald , “Monitoring Thyroid Function in Patients on Levothyroxine. Assessment of Conformity to National Guidance and Variability in Practice,” Experimental and Clinical Endocrinology & Diabetes 125, no. 9 (2017): 625–633. 28407667 10.1055/s-0043-103018

[25] https://bnf.nice.org.uk/drugs/levothyroxine‐sodium. Accessed 2 July 2025.

[26] V. Calsolaro , F. Niccolai , G. Pasqualetti , et al., “Hypothyroidism in the Elderly: Who Should be Treated and How?,” Journal of the Endocrine Society 3, no. 1 (2018): 146–158. 30607373 10.1210/js.2018-00207 PMC6309133

[27] M. Lillevang‐Johansen , B. Abrahamsen , H. L. Jørgensen , T. H. Brix , and L. Hegedüs , “Duration of Over‐ and Under‐Treatment of Hypothyroidism Is Associated With Increased Cardiovascular Risk,” European Journal of Endocrinology 180, no. 6 (2019): 407–441. 31035256 10.1530/EJE-19-0006

[28] D. Khandelwal and N. Tandon , “Overt and Subclinical Hypothyroidism: Who to Treat and How,” Drugs 72, no. 1 (2012): 17–33. 22191793 10.2165/11598070-000000000-00000

[29] L. Ingoe , N. Phipps , G. Armstrong , A. Rajagopal , F. Kamali , and S. Razvi , “Prevalence of Treated Hypothyroidism in the Community: Analysis From General Practices in North‐East England With Implications for the United Kingdom,” Clinical Endocrinology 87, no. 6 (2017): 860–864. 28782887 10.1111/cen.13440

[30] https://cks.nice.org.uk/topics/hypothyroidism/background‐information/prevalence. Accessed 2 July 2025.

[31] A. H. Heald , P. Taylor , L. Premawardhana , M. Stedman , and C. Dayan , “Natural Desiccated Thyroid for the Treatment of Hypothyroidism?,” Front Endocrinol (Lausanne) 8, no. 14 (2024): 1309159. 10.3389/fendo.2023.1309159 PMC10801060 38260143

[32] A. Heald , M. Livingston , and D. Hughes , “Management of Patients Symptomatically Unresponsive to Levothyroxine: Natural Desiccated Thyroid Extract or the Combination of Levothyroxine and Liothyronine? A Research Priority,” Experimental and Clinical Endocrinology & Diabetes 128, no. 9 (2020): 596–598, 10.1055/a-1062-6167. 31820425

[33] P. Saravanan , W. F. Chau , N. Roberts , K. Vedhara , R. Greenwood , and C. M. Dayan , “Psychological Well‐Being in Patients on ‘Adequate’ Doses of l‐Thyroxine: Results of a Large, Controlled Community‐Based Questionnaire Study,” Clinical Endocrinology 57 (2002): 577–585. 12390330 10.1046/j.1365-2265.2002.01654.x

[34] V. Panicker , J. Evans , T. Bjøro , B. O. Åsvold , C. M. Dayan , and O. Bjerkeset , “A Paradoxical Difference in Relationship Between Anxiety, Depression and Thyroid Function in Subjects on and Not on T4: Findings From the HUNT Study,” Clinical Endocrinology 71 (2009): 574–580. 19751298 10.1111/j.1365-2265.2008.03521.x

[35] A. Heald , L. D. Premawardhana , P. N. Taylor , et al., “Liothyronine (LT3) Prescribing in England: Are Cost Constraints Inhibiting Guideline Implementation?,” Clinical Endocrinology 101, no. 1 (2024): 62–68. 38752469 10.1111/cen.15061

[36] N. F. Dirks , W. P. J. den Elzen , J. J. Hillebrand , et al., “Should We Depend on Reference Intervals From Manufacturer Package Inserts? Comparing TSH and FT4 Reference Intervals From Four Manufacturers With Results From Modern Indirect Methods and the Direct Method,” Clinical Chemistry and Laboratory Medicine 62, no. 7 (2024): 1352–1361. 38205847 10.1515/cclm-2023-1237

[37] P. N. Taylor , A. Iqbal , C. Minassian , et al., “Falling Threshold for Treatment of Borderline Elevated Thyrotropin Levels‐Balancing Benefits and Risks: Evidence From a Large Community‐Based Study,” JAMA Internal Medicine 174 (2014): 32–39. 24100714 10.1001/jamainternmed.2013.11312

[38] H. L. Duce , C. J. Duff , S. Zaidi , C. Parfitt , A. H. Heald , and A. A. Fryer , “Evaluation of Thyroid Function Monitoring in People Treated With Lithium: Advice Based on Real‐World Data,” Bipolar Disorders 25, no. 5 (2023): 402–409. 36645255 10.1111/bdi.13298

[39] M. Stedman , P. Taylor , I. Halsall , et al., “Evaluation of Thyroid Hormone Status: Are Simultaneous TSH and FT4 Tests Necessary? Analysis of Thyroid Function Test Results Taken From the Greater Manchester Care Record 2010‐2023,” Clinical Endocrinology 103, no. 6 (2025): 906–908. 40827564 10.1111/cen.70012 PMC12583302

